# Effects of changes in inspired oxygen fraction on urinary oxygen tension measurements

**DOI:** 10.1186/s40635-022-00479-y

**Published:** 2022-12-12

**Authors:** Eduardo A. Osawa, Salvatore L. Cutuli, Fumitaka Yanase, Naoya Iguchi, Laurent Bitker, Alexandre T. Maciel, Yugeesh R. Lankadeva, Clive N. May, Roger G. Evans, Glenn M. Eastwood, Rinaldo Bellomo

**Affiliations:** 1 Imed Group Research Department, Sao Paulo, Brazil; 2 Intensive Care Unit, Hospital Sao Camilo, Unidade Pompeia, Sao Paulo, Brazil; 3 Dipartimento di Scienze dell’Emergenza, Anestesiologiche e della Rianimazione, Fondazione Policlinico Universitario A. Gemelli IRCCS, Rome, Italy; 4 Università Cattolica del Sacro Cuore, Rome, Italy; 5 Department of Intensive Care, Austin Hospital, Melbourne, VIC 3084 Australia; 6 Australian and New Zealand Intensive Care Research Centre, Monash University, Melbourne, Australia; 7 Department of Anaesthesiology and Intensive Care Medicine, Graduate School of Medicine, Osaka University, Suita, Japan; 8 Service de Médecine Intensive – Réanimation, Hôpital de La Croix Rousse, Hospices Civils de Lyon, Lyon, France; 9 Pre-Clinical Critical Care Unit, Florey Institute of Neuroscience and Mental Health, University of Melbourne, Melbourne, VIC Australia; 10 Department of Critical Care, Melbourne Medical School, University of Melbourne, Melbourne, VIC Australia; 11 Cardiovascular Disease Program, Biomedicine Discovery Institute and Department of Physiology, Monash University, Melbourne, Australia

**Keywords:** Inspired oxygen fraction, Sepsis, Septic shock, Urinary oxygen tension, Urinary oxygenation

## Abstract

**Background:**

Continuous measurement of urinary PO_2_ (PuO_2_) is being applied to indirectly monitor renal medullary PO_2_. However, when applied to critically ill patients with shock, its measurement may be affected by changes in FiO_2_ and PaO_2_ and potential associated O_2_ diffusion between urine and ureteric or bladder tissue. We aimed to investigate PuO_2_ measurements in septic shock patients with a fiberoptic luminescence optode inserted into the urinary catheter lumen in relation to episodes of FiO_2_ change. We also evaluated medullary and urinary oxygen tension values in Merino ewes at two different FiO_2_ levels.

**Results:**

In 10 human patients, there were 32 FiO_2_ decreases and 31 increases in FiO_2_. Median pre-decrease FiO_2_ was 0.36 [0.30, 0.39] and median post-decrease FiO_2_ was 0.30 [0.23, 0.30], *p* = 0.006. PaO_2_ levels decreased from 83 mmHg [77, 94] to 72 [62, 80] mmHg, *p* = 0.009. However, PuO_2_ was 23.2 mmHg [20.5, 29.0] before and 24.2 mmHg [20.6, 26.3] after the intervention (*p* = 0.56). The median pre-increase FiO_2_ was 0.30 [0.21, 0.30] and median post-increase FiO_2_ was 0.35 [0.30, 0.40], *p* = 0.008. PaO_2_ levels increased from 64 mmHg [58, 72 mmHg] to 71 mmHg [70, 100], *p* = 0.04. However, PuO_2_ was 25.0 mmHg [IQR: 20.7, 26.8] before and 24.3 mmHg [IQR: 20.7, 26.3] after the intervention (*p* = 0.65). A mixed linear regression model showed a weak correlation between the variation in PaO_2_ and the variation in PuO_2_ values. In 9 Merino ewes, when comparing oxygen tension levels between FiO_2_ of 0.21 and 0.40, medullary values did not differ (25.1 ± 13.4 mmHg vs. 27.9 ± 15.4 mmHg, respectively, *p* = 0.6766) and this was similar to urinary oxygen values (27.1 ± 6.17 mmHg vs. 29.7 ± 4.41 mmHg, respectively, *p* = 0.3192).

**Conclusions:**

Changes in FiO_2_ and PaO_2_ within the context of usual care did not affect PuO_2_. Our findings were supported by experimental data and suggest that PuO_2_ can be used as biomarker of medullary oxygenation irrespective of FiO_2_.

**Supplementary Information:**

The online version contains supplementary material available at 10.1186/s40635-022-00479-y.

## Background

Critically ill patients with sepsis develop acute kidney injury (AKI) in 20 to 50% of cases [1]. Despite its clinical importance, available methods for early detection of kidney damage have major limitations. One of the mechanisms implicated in the pathophysiology of this condition is hypoxia of renal tissue, particularly in the renal medulla [2]. Selective hypoxia in the renal medulla was observed in an ovine model of sepsis, despite the presence of global renal hyperemia [3]. In clinical practice, it is not feasible to measure renal medullary tissue oxygen tension in the intensive care unit (ICU). Nevertheless, it is possible to measure oxygen tension of bladder urine (PuO_2_) by the introduction of a fiber-optic probe in the bladder catheter [4, 5]. A number of experimental investigations have been performed to evaluate this technique and have documented a robust correlation between urinary PO_2_ and medullary PO_2_ [6].

One of the caveats for the understanding of relationship between urinary and medullary PO_2_ is that it may be cofounded by several factors. A concern arising from experimental and computational models is that systemic arterial oxygen tension (PaO_2_) may affect ureteric and bladder wall oxygenation which, in turn, may influence PuO_2_ [7–10]. Experimental observations in anesthetized rabbits suggested that oxygen exchange within the urinary tract is slow and is unlikely to be a major confounder of the relationship between renal medullary tissue PO_2_ and PuO_2_ [7]. Nevertheless, the potential for such confounding in human sepsis, where PuO_2_ measurement might be applied to guide management of risk of AKI and therapeutic interventions, remains unknown. Therefore, to assess whether systemic oxygenation has a potentially confounding impact on urinary oxygenation, we aimed to evaluate PuO_2_ in critically ill patients with sepsis during the periods before and after changes in fractional inspired oxygen (FiO_2_) instituted to manage PaO_2_ within clinically acceptable levels. Also, to support our clinical findings, we investigated the medullary and urinary oxygen tension levels in a sheep experiment within a similar range of FiO_2_ variation.

## Methods

### Observational study in septic patients

#### Study design

We performed a prospective observational cohort study in the ICU of a tertiary care hospital located in Melbourne, Australia, from January 2017 to March 2018. The protocol was approved by the Human Research Ethics Committee of the Austin Hospital (HREC/16/Austin/26). Written informed consent was obtained from all participants or their legal representatives.

#### Participants

A convenience sample of adult patients (18 years old or older) with suspected or confirmed septic shock was enrolled in the study. We excluded patients who were anuric, on chronic dialysis, pregnant, or who were kidney transplant recipients.

#### *Measurement of PuO*_*2*_

For each patient, a fiberoptic luminescence optode (NX-LAS-1/O/E-5 m, Oxford Optronix, Abingdon, UK) was inserted into the lumen of the urinary catheter via a sterile procedure, as described in detail previously [11]. In brief, the sensing tip of the probe was advanced to the distal tip of the catheter so that the probe was placed inside the bladder. The fiberoptic probe was connected to a luminescence lifetime oximeter (Oxylite Pro, Oxford Optronix, UK) interfaced with a laptop computer running LabChart software (Version 8, ADInstruments, Bella Vista, NSW, Australia). PuO_2_ was recorded every minute from the time of the probe insertion until the removal of the urinary catheter by the treating medical team or at ICU discharge, whichever occurred first.

#### *FiO*_*2*_* settings and measurement of PaO*_*2*_* and PuO*_*2*_

**Fig. 1:**
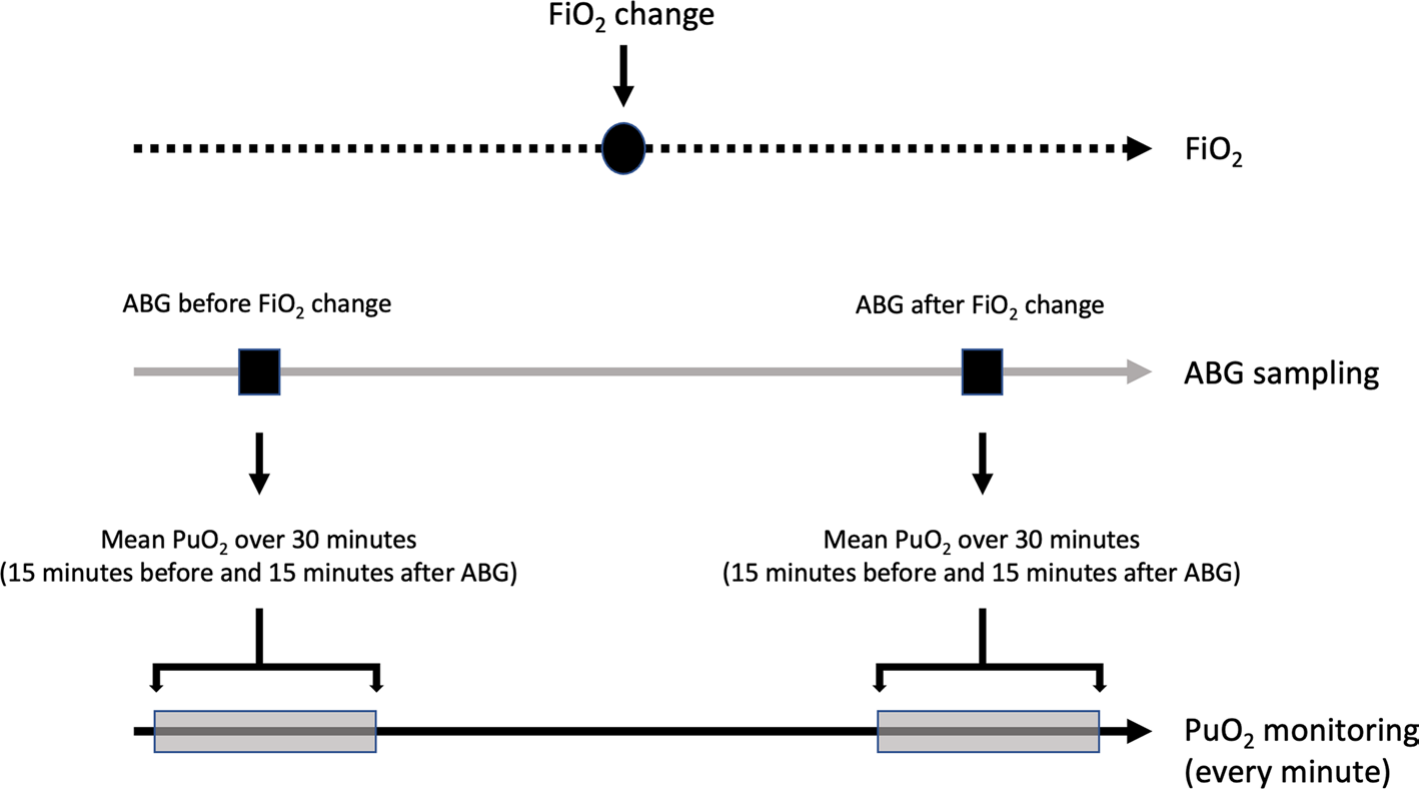
Schematic representation of the procedure used to obtain PaO_2_ and PuO_2_ measurements before and after an episode of FiO_2_ change during the observation period. ABG: arterial blood gas; FiO_2_: fraction of inspired oxygen; PuO_2_: urinary oxygen tension

FiO_2_ was modified at the discretion of bedside clinicians to maintain a peripheral oxygen saturation level greater than 90%. Episodes of FiO_2_ change were documented in the laptop computer software by the bedside nurse at the moment the intervention took place and were verified against observation charts. To describe arterial oxygen levels, we identified arterial blood gases (ABG) that were collected before and after FiO_2_ modification (Fig. 1). To make before and after periods distinctive, we restricted the analysis to ABG samples that were obtained within a time difference from FiO_2_ change of 30 min or more. For each PaO_2_ measurement, we obtained the mean value of 30 PuO_2_ measurements centerd around the exact time when the ABG was collected (15 min before and 15 min after the blood was drawn). Blood gas analysis was performed with an ABL800 FLEX blood gas machine (Radiometer, Copenhagen, Denmark). No specific method was used to ascertain the ABG stability for the purposes of the study. However, the unit where the study was conducted is a world-class intensive care with a 1:1 nurse-to-patient ratio. The ABG analyzer is located inside the unit and the team is trained to obtain reliable measurements according to the institutional protocol.

#### Data extraction

Aside from the abovementioned variables, we collected information on age, gender, baseline creatinine, infection source, comorbidities and ICU severity scores. We also recorded data on duration of mechanical ventilation, number of hours in mandatory and spontaneous modes, mechanical ventilation parameters, end-tidal partial pressure of carbon dioxide (EtCO_2_), ICU and hospital length of stay, and hospital mortality.

### Experimental study using healthy adult sheep

#### Animal preparation

We obtained data from 9 healthy adult Merino ewes included in an experimental study of sheep undergoing aseptic surgical procedures under general anesthesia [18]. The study was approved by the Animal Ethics Committee of the Florey Institute of Neuroscience and Mental Health under guidelines laid down by the National Health and Medical Research Council of Australia. A similar fiberoptic luminescence optode (Oxford Optronix, Abingdon, UK) used in human patients was inserted into the lumen of the urinary catheter and surgically inserted into the renal medulla. The tissue oxygen tension was continuously recorded at 100 Hz on a computer using a CED micro 1401 interface with Spike 2 software (Cambridge Electronic Design, Cambridge, United Kingdom).

#### *Experimental protocol for the variation of FiO*_*2*_

The protocol had 4 components of 20-min duration: a 10-min period to allow oxygen levels to stabilize followed by a 10-min experimental period. Our primary experimental stabilization criteria included renal medullary PO_2_ and urinary PO_2_. This timing was determined by assessing the medullary and urinary PO_2_ values over time. A block randomization was used to set FiO_2_ at 0.21, 0.40, 0.60 and 1.0. The total gas flow on the mechanical ventilator was maintained at a constant rate of 1.5 L/min, whilst the ratio of the individual oxygen-to-air gas volumes was altered to achieve the target FiO_2_. For the current analysis, we obtained data from the periods when FiO_2_ levels were 0.21 and 0.40.

### Statistical analyses

Continuous variables are reported as median (quartile 1, quartile 3) and categorical variables are reported as number (%). An aggregate measure was calculated per patient and the paired-sample Wilcoxon test was used to compare median values between the two time periods (before and after FiO_2_ change). Proportions were compared using Fisher’s exact test. To account for multiple episodes of FiO_2_ change per patient, we performed a mixed linear regression model to assess the relationship between the variation of PaO_2_ (ΔPaO_2_) and the variation of PuO_2_ (ΔPuO_2_).

In the sheep experiment, values for each FiO_2_ setting were calculated and a comparison between groups was performed by using Kruskal–Wallis test.

Statistical analysis was performed using R version 4.0.5. Two-tailed *p* ≤ 0.05 was considered statistically significant.

## Results

### Human septic patients

**Fig. 2:**
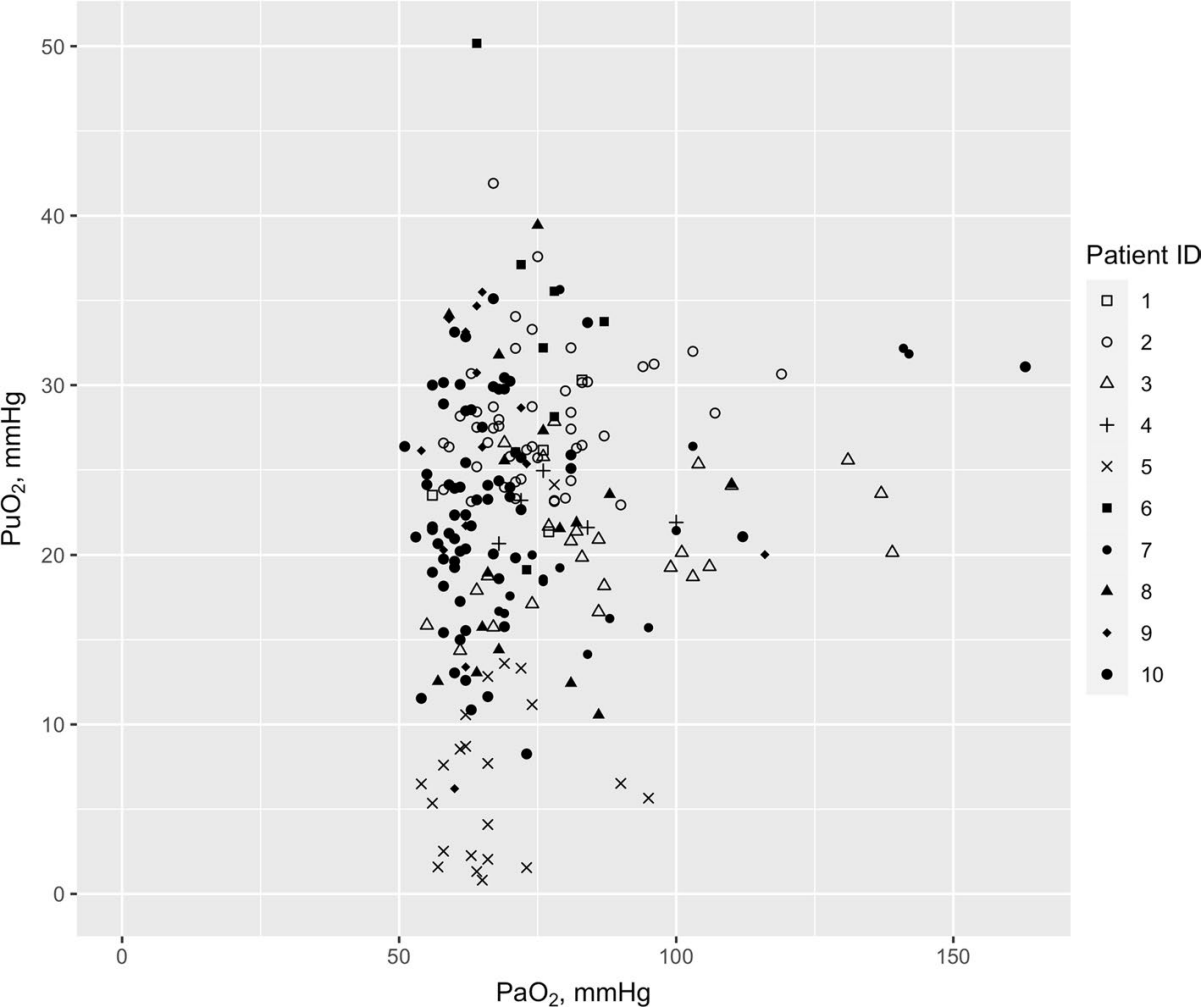
Scatterplot of arterial and urinary oxygen tension measured in the ten patients enrolled in the study. PuO_2_: urinary oxygen tension; PaO_2_: arterial blood oxygen tension

We studied 10 patients, whose clinical characteristics are reported in Table 1. During the study period, patients were mechanically ventilated for 733 h (88.9% of total duration), of which 459 h were in spontaneous mode (55.6%). The mechanical ventilation parameters during the study period are described in Table 2. Arterial blood gases were obtained in 233 occasions. Across the cohort of 10 patients there was a weak but statistically significant positive association between PaO_2_ and PuO_2_ (Fig. 2, *r*^2^ = 0.022, *p* = 0.004). This relationship was plotted for each patient (Additional file 1: Fig. S1).

**Table 1:** Baseline characteristics of study patients

Patient number	Age	Gender	Baseline creatinine	Infection source	Comorbidities	APACHE II	APACHE III	Death	ICU LOS (days)	Hospital LOS (days)	Episodes of FiO_2_ increase	Episodes of FiO_2_ decrease
1	45	Male	67	Unknown	None	10	28	No	12	18	1	1
2	80	Female	159	Ventilator-associated pneumonia	Post emergent left artery embolectomy for ischemic lower limb	19	71	No	8	18	10	8
3	53	Female	88	Ischemic ileal perforation	Post-laparotomy, hepatitis C cirrhosis	16	41	No	4	28	3	3
4	74	Male	335	Biliary	Cirrhosis, IHD, AF, AS, gout	33	113	Yes	4	4	1	1
5	68	Female	64	Pneumonia	Diabetes, alcohol abuse, depression	31	111	Yes	18	18	5	3
6	55	Female	47	Pneumonia	Diabetes, smoking	16	37	No	5	26	1	2
7	76	Female	68	Pneumonia	Diabetes, AF, hypertension	25	105	No	8	22	0	2
8	50	Female	76	Viral pneumonia	Oesophageal reflux	10	39	No	4	9	1	3
9	56	Female	47	Necrotizing pneumonia with empyema	Wegener's granulomatosis, COPD, leg necrotic ulcer	27	74	No	7	22	1	2
10	69	Male	88	Influenza A	COPD, peripheral vascular disease, OSA	22	44	No	25	31	8	7

APACHE: Acute and Chronic Health Evaluation; LOS: length of stay; IHD: ischemic heart disease; AF: atrial fibrillation; AS: aortic stenosis; COPD: chronic obstructive pulmonary disease; OSA: obstructive sleep apnea

**Table 2:** Mechanical ventilation parameters of human patients

Parameter	
Number of hours in mandatory MV mode, *n* (%)	274 (33.2%)
Number of hours in spontaneous MV mode, *n* (%)	459 (55.6%)
Number of hours not in MV, *n* (%)	92 (11.2%)
Tidal volume, ml	450 (400, 550)
Inspiratory pressure, cmH_2_O, median (IQR)	18 (15, 22)
Respiratory rate, breaths per minute, median (IQR)	17 (13, 21)
Pressure support, cmH_2_O, median (IQR)	10 (10, 14)
PEEP, cmH_2_O, median (IQR)	5 (5, 8)
Minute-volume, L/min, median (IQR)	7.7 (6.7, 9.5)
EtCO2, mmHg, median (IQR)	39 (34, 50)

EtCO2: end-tidal carbon dioxide; MV: mechanical ventilation

We observed 63 episodes of changes in the FiO_2_ setting: on 32 occasions FiO_2_ was decreased and on 31 FiO_2_ was increased. In the episodes where FiO_2_ decreased, the median [Q1, Q3] pre-intervention FiO_2_ was 0.36 [0.30, 0.39] and the median post-intervention FiO_2_ was 0.30 [0.23, 0.30] (*p* = 0.006). When FiO_2_ increased, the median FiO_2_ before the intervention was 0.30 [0.21, 0.30] and the median FiO_2_ after the intervention was 0.35 [0.30, 0.40], *p* = 0.008. There were 14 episodes of successive increase/decrease in the same patient. Such episodes were successive and conditional on the presence of a PaO_2_ measurement and the time difference between these episodes was 4.29 [2.49, 5.38] hours.

**Fig. 3:**
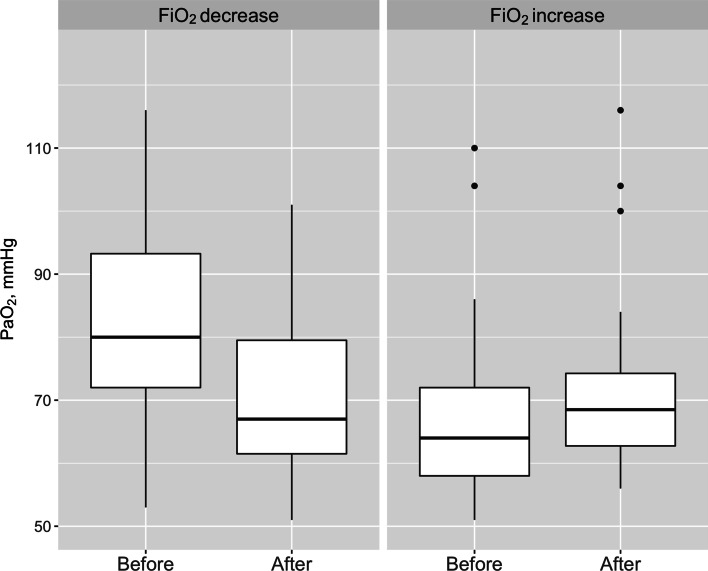
Box plot representation of arterial blood oxygen tension before and after changes in fractional inspired oxygen. FiO_2_: fractional inspired oxygen; PaO_2_: arterial blood oxygen tension

**Fig. 4:**
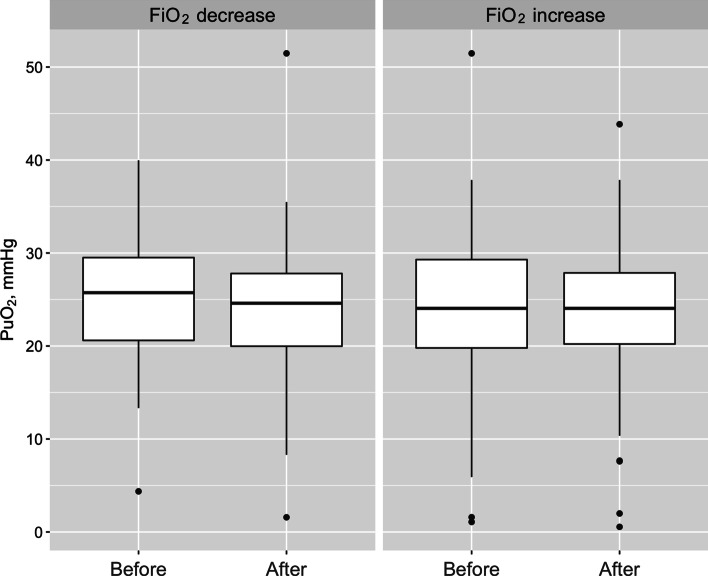
Box plot representation of urinary oxygen tension before and after changes in fractional inspired oxygen. FiO_2_: fractional inspired oxygen; PuO_2_: urinary oxygen tension

In the episodes when FiO_2_ was decreased, PaO_2_ fell from 83 [77, 94] mmHg to 72 [62, 80] mmHg (*p* = 0.009, Fig. 3). Nevertheless, PuO_2_ did not vary significantly across the two time points, being 23.2 [20.5, 29.0] mmHg before the intervention and 24.2 [20.6, 26.3] mmHg after the intervention (*p* = 0.557, Fig. 4). In such episodes, ΔPaO_2_ was − 14 [− 22.2, − 3.0] mmHg and ΔPuO_2_ was − 0.02 [− 4.3, 2.9] mmHg.

**Fig. 5:**
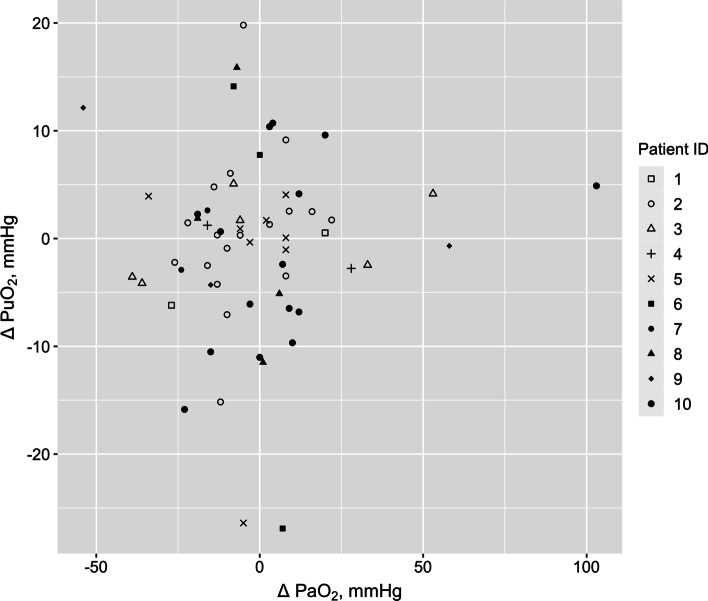
Relationship between the changes in arterial oxygen tension (Δ PaO_2_) and urinary oxygen tension (Δ PuO_2_)

When FiO_2_ was increased, PaO_2_ increased from 64 [58, 72] mmHg to 71 [70, 100] mmHg (*p* = 0.038, Fig. 3). The corresponding PuO_2_ measurements were 25.0 [20.7, 26.8] mmHg before the intervention and 24.3 [20.7, 26.3] mmHg after the intervention (*p* = 0.652, Fig. 4). ΔPaO_2_ was 8 [− 5.5, 14.0] mmHg and ΔPuO_2_ was 0.5 [− 2.6, 4.1] mmHg. A mixed linear regression model showed a weak relationship between the change in PaO_2_ and the change in PuO_2_ (*r*^2^ = 0.003, *p* = 0.652, Fig. 5) Also, we obtained of an aggregated measure per patient and observed that ΔPuO_2_ was − 0.532 [− 1.410, 0.331] mmHg and ΔPaO_2_ was − 3.25 [− 8.880, − 0.125] mmHg, *p* = 0.1431.

Other laboratory parameters measured before and after FiO_2_ change were similar between the two time points (Table 3). The urine output before FiO_2_ change was 63.8 [32.5, 95.0] ml and 70.5 [30.6, 95.0] after FiO_2_ change, *p* = 0.9396 (Additional file 2: Fig. S2).

**Table 3:** Laboratory values before and after FiO2 change in human patients

	Before	After	*p* value
FiO2 decrease			
pH	7.41 [7.38, 7.46]	7.41 [7.39, 7.45]	0.722
pCO2	41.50 [36.75, 46.5]	42.75 [36.5, 45.63]	0.734
Bicarbonate	27.00 [22.88, 29.88]	27.00 [23.25, 30.25]	0.098
Base excess	3.00 [0, 5.00]	2.50 [− 0.75, 5.75]	0.335
Chloride	103.50 [102.00,106.88]	103.50 [101.63, 106.88]	1.000
Lactate	1.28 [1.03, 1.88]	1.35 [1.01, 2.01]	1.000
Creatinine	67.00 [59.00, 80.00]	70.50 [58.13, 80.88]	1.000
Hemoglobin	92.50 [79.80, 119.00]	94.25 [78.25, 118.13]	0.888
Glucose	9.65 [6.38, 11.55]	10.25 [7.53, 11.05]	0.529
FiO_2_ increase			
pH	7.41 [7.39, 7.45]	7.42 [7.36, 7.43]	1.000
pCO_2_	43.00 [35.00, 44.50]	44.00 [34.00, 45.50]	1.000
Bicarbonate	26.00 [24.00, 27.00]	27.00 [22.00, 28.00]	0.583
Base excess	2.00 [0, 3.00]	2.00 [0, 4.00]	0.684
Chloride	106.00 [103.00, 109.00]	107.00 [103.00, 107.00]	0.833
Lactate	1.60 [1.00, 1.90]	1.60 [1.10, 3.00]	0.176
Creatinine	65.50 [58.88, 70.75]	66.50 [62.75, 75.25]	0.178
Hemoglobin	103.00 [79.00, 117.00]	102.00 [80.00, 127.00]	0.344
Glucose	9.90 [8.50, 10.90]	10.30 [7.80, 11.70]	0.910

### Experimental study

In the sheep experiment, we evaluated urinary and medullary tissue oxygen measurements in four FiO_2_ levels: 0.21, 0.40, 0.60 and 1.00 (Table 4). For each variable, a total of 36 measurements were obtained. The median PaO_2_ value at 0.21 FiO2 was 54.5 [51.3, 74.4] mmHg, 209 [181, 223] mmHg at 0.40 FiO2, *p* < 0.001. When comparing 0.21 and 0.40 FiO_2_, we found no statistically significant difference in oxygen tension measurements. The medullary oxygen tension was 25.3 [15.3, 30.5] mmHg at 0.21 FiO_2_ and 28.3 [15.9, 43.4] mmHg at 0.40 FiO_2_, *p* = 0.6766; and the urinary oxygen tension was 25.5 [21.6, 32.6] mmHg at 0.21 FiO_2_ and 30.0 [27.4, 33.6] mmHg at 0.40 FiO_2_, *p* = 0.3192.

**Table 4:** Measurements of PaO_2_, medullary oxygen tension and urinary oxygen tension under different FiO_2_ settings obtained in the experimental study using healthy adult sheep

Variable	0.21 FiO_2_	0.40 FiO_2_	0.60 FiO_2_	1.00 FiO_2_	*p*-value
Medullary oxygen tension, mmHg	25.3 [15.3, 30.5]	28.3 [15.9, 43.4]	33.4 [22.6, 45.0]	40.0 [34.9, 46.8]	0.087
Urinary oxygen tension, mmHg	25.5 [21.6, 32.6]	30.0 [27.4, 33.6]	59.4 [36.5, 66.0]	87.9 [66.1, 99.8]	< 0.001
PaO_2_, mmHg	54.5 [51.3, 74.4]	209 [181, 223]	303 [282, 306]	510 [499, 525]	< 0.001

PaO_2_: end-tidal carbon dioxide; MV: mechanical ventilation

At higher FiO_2_ levels, PaO_2_ values increased (303 [282, 306] mmHg at 0.60 FiO2 and 510 [499, 525] mmHg at 1.00 FiO_2_. Furthermore, medullary oxygen tension tended to increase at these levels (33.4 [22.6, 45.0] mmHg at 0.60 FiO_2_ and 40.0 [34.0, 46.8] mmHg at 1.00 FiO_2_ (*p* = 0.087) while urinary oxygen tension values significantly increased (59.4 [36.5, 66.0] mmHg at 0.60 FiO_2_ and 87.9 [66.1, 99.8] mmHg at 1.00 FiO_2_ (*p* < 0.001).

For a larger change in FiO_2_ (from 0.21 to 0.60), medullary oxygen tension values were similar (25.3 [15.3, 30.5] mmHg vs. 33.4 [22.6, 45.0] mmHg, *p* = 0.2224) but urinary oxygen tension values increased (25.5 [21.6, 32.6] mmHg vs. 59.4 [36.5, 66.0] mmHg, *p* = 0.001).

## Discussion

### Key findings

We conducted an observational study in human septic patients to determine whether PuO_2_ is affected by changes in systemic oxygenation during routine care of patients with septic shock. As expected, changes of FiO_2_ resulted in significant changes in PaO_2_. However, we found no significant differences between PuO_2_ measured before and after the interventions occurred. We supported our clinical findings with data from an experimental sheep experiment showing that medullary and urinary oxygen tension measurements did not differ within a similar range of FiO_2_ variation.

### Relationship to previous studies

To our knowledge, there have been no previous investigations of the relationship between systemic and urinary oxygenation in human patients with septic shock. Ngo et al. addressed this issue in a group of patients undergoing cardiac surgery, finding no significant relationship between PaO_2_ and PuO_2_ during cardiopulmonary bypass [6]. Importantly, however, these observations were obtained in a unique physiological state with non-pulsatile flow, an extracorporeal circuit and mild hypothermia. Our current observations are likely more generally applicable to patients in a critical care setting. They are also very consistent with the outcomes of a retrospective analysis of three 3 studies involving a total of 28 adult Merino ewes during experimental sepsis [4, 12, 13], in which only a weak linear relationship was found between PaO_2_ and PuO_2_, accounting for ≤ 6% of the variation of PuO_2_ [6].

The absence of detectable changes in PuO_2_ in response to modest but clinically significant changes in FiO_2_ and thus PaO_2_ indicate that renal medullary tissue PO_2_ was not markedly affected by these clinical maneuvers. Experimental evidence supports the concept that extreme variations in FiO_2_ and/or PaO_2_ lead to corresponding changes in the oxygen tension of renal tissue. For example, in anesthetized rats a reduction in FiO_2_ from 1.0 to 0.1 resulted in a decline in cortical and medullary microvascular PO_2_ as assessed by dual-wavelength phosphorimetry [14]. Likewise, studies using fluorescence optodes in anesthetized rabbits demonstrated variations in both cortical and medullary tissue PO_2_ with variations in FiO_2_ [7, 15–17]. These findings provide support to our experimental findings that greater FiO_2_ variation is associated with greater PuO_2_ response, in particular at 0.60 and 1.00 FiO_2_.

Our ovine study sample derived from a larger experiment comprising 18 healthy sheep undergoing abdominal surgery under total intravenous or volatile anesthesia. In this study, increasing FiO_2_ from 0.21 to 1.00 increased cortical and medullary tissue PO_2_ [18]. However, it is also well-established that renal medullary tissue PO_2_ is less responsive to changes in PaO_2_ than the renal cortical tissue PO_2_ [16, 18, 19]. This appears to be a consequence of counter-current diffusive shunting of oxygen between descending and ascending vasa recta, which acts to reduce delivery of oxygen to renal medullary tissue [20]. Consequently, small but physiologically (and clinically) significant changes in FiO_2_ and/or PaO_2_ may not appreciably alter renal medullary tissue PO_2_. In support of this concept, no appreciable difference was observed in medullary tissue PO_2_ in our group’s preceding experiment when FiO_2_ was varied from 0.4 to 0.6 [18]. Similarly, in anesthetized rats outer medullary microvascular PO_2_ did not vary significantly when FiO_2_ was varied from 0.21 and 0.30 [21]. We cannot directly measure renal medullary tissue PO_2_ in patients and can only draw indirect inferences from measurement of PuO_2_ and consideration of available experimental evidence. However, the most parsimonious interpretation of our current findings is that modest changes in FiO_2_ and thus PaO_2_ neither markedly alter renal medullary tissue PO_2_ in patients with sepsis nor confounded the relationship between medullary tissue PO_2_ and PuO_2_.

### Study implications

Our findings suggest that commonly performed adjustments to FiO_2_ settings in patients with sepsis do not result in significant changes in PuO_2_. In consonance of these findings, we observed that variations of FiO_2_ between 0.21 and 0.40 did not alter either medullary or urinary oxygen tension measurements in a sheep experiment. Thus, variations of systemic oxygenation seem unlikely to confound or affect the utility of urinary oxygenation as a biomarker for risk of AKI. Nevertheless, at higher FiO_2_ (0.60 and 1.00), significantly increased PuO_2_ values were obtained. One possible explanation is that in our septic patients, the FiO_2_ gap was far smaller in comparison to the experimental study. Also, one could argue that a type 2 error was present in the observational study which may have been controlled for during the experimental protocol.

Additional investigation is needed to explore whether the lack of PuO_2_ variation in face of PaO_2_ changes derives from the presence of confounding factors affecting medullary oxygen values. Also, further studies in critically ill patients are needed to elucidate whether sustained differences in oxygen exposure [22, 23] influence renal related outcomes. Thus, changes in PaO_2_, as a consequence of altered FiO_2_ in routine care of patients with septic shock, is unlikely to be a major confounder of the relationship between renal medullary tissue PO_2_ and PuO_2_. In the current study, PaO_2_ was used as a measure of systemic oxygenation because it reflects the balance between oxygen delivery and consumption. Had SpO_2_ been used, the accuracy would have been affected by peripheral tissue perfusion, use of vasoactive agents and altered cardiac output. Finally, continuous measurement of PuO_2_ might be useful for monitoring the impact on renal medullary oxygenation.

### Strengths and limitations

We evaluated systemic and urinary oxygenation in human septic patients and assisted our proposition with experimental data. Our findings are consistent with previous observations in sepsis [6] and provide additional evidence that the relationship between renal medullary tissue PO_2_ and PuO_2_ is unlikely to be confounded by changes in FiO_2_ or PaO_2_ in the range commonly encountered in the ICU. As such, they provide further support for the use of PuO_2_ as a clinical surrogate of renal medullary PO_2_.

Our study has several limitations. First, the clinical component was an observation designed to assess the effects on PuO_2_ where the observed intervention (change of FiO_2_) was not protocolized. Moreover, controlling for variables such as creatinine or urine output was not feasible due to technical limitations and the limited number of patients. However, we aimed to undertake an exploratory analysis to generate a preliminary hypothesis to guide advanced studies. Moreover, we added data from a sheep experiment where FiO_2_ variation was protocolized. Also, due to the lower number of measurements in the experimental study, greater heterogeneity was observed. Second, the inclusion of septic patients in our clinical study did not occur in the early stage of resuscitation. On the other hand, the instances of FiO_2_ change we captured took place in a stable state with lower propensity for PuO_2_ to be affected by additional confounding effects of interventions intended to optimize oxygen delivery to the tissues. Furthermore, changes in FiO_2_ performed under stable conditions might have reduced the likelihood of reverse causation or provided mitigation of any potential effect of other interventions. Third, the observational nature of the study may have led to confounding by indication. For instance, the reasons motivating the clinician to change FiO_2_ settings could have affected the relationship between FiO_2_ and PuO_2_. However, a larger degree of FiO_2_ change would be expected if optimization measures capable of affecting such relationship were in place. Our patients were enrolled in the stabilization phase of sepsis, a time when, in general, only limited interventions are performed to achieve physiologic parameters aiming to prevent organ dysfunction. Finally, we addressed only the variation of systemic oxygenation within the normoxemic range. However, such a normoxemic range is typical in the care of patients in the ICU.

## Conclusions

Changes in FiO_2_ and PaO_2_ within the context of usual care did not appreciably affect PuO_2_. Our findings suggest that, within the values reported, PuO_2_ measured in a clinical and experimental setting is not confounded by changes in inspired oxygen fraction or arterial oxygen tension and that PuO2 can be used as biomarker of medullary oxygenation irrespective of FiO2.

## Supplementary Information

**Additional file 1.** Relationship between PaO_2_ and PuO_2_ values in each individual study patient.

**Additional file 2.** Box plot illustrating the hourly urinary output before and after a change in FiO_2_.

## Data Availability

Data are available by contacting the corresponding author.

## Abbreviations

ABG: Arterial blood gas test
AKI: Acute kidney injury
EtCO_2_: End-tidal partial pressure of carbon dioxide
FiO_2_: Fraction of inspired oxygen.
ICU: Intensive Care Unit
PaO_2_: Arterial oxygen tension
PuO_2_: Urinary oxygen tension
