# Supplementary figures and images for: Effects of changes in inspired oxygen fraction on urinary oxygen tension measurements
Source: Intensive Care Med Exp. 2022 Dec 12;10:52. doi: 10.1186/s40635-022-00479-y (PMC9742069; doi:10.1186/s40635-022-00479-y)

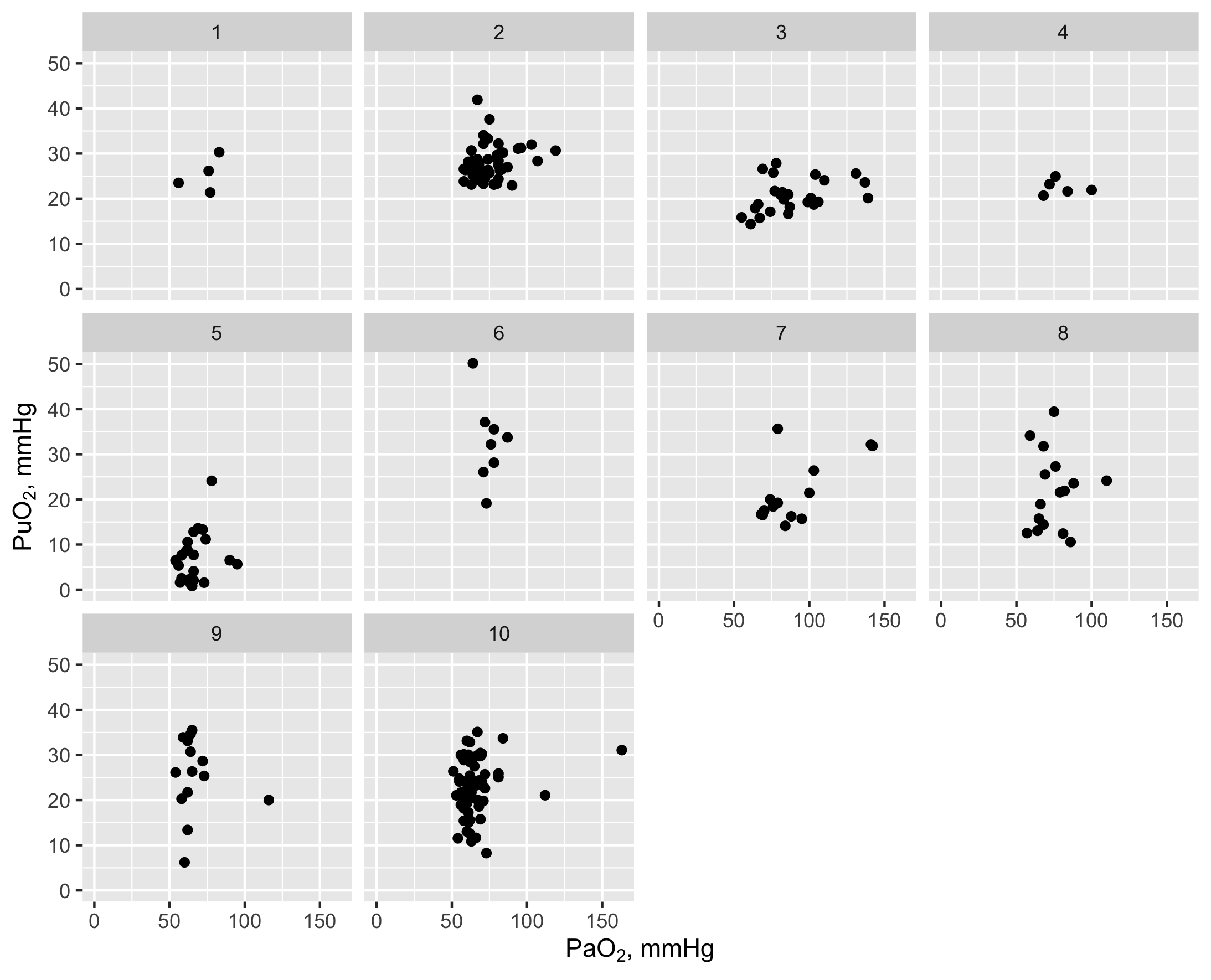

Supplement: Supplementary file 1 — Additional file 1. Relationship between PaO2 and PuO2 values in each individual study patient. [file 40635_2022_479_MOESM1_ESM.png]

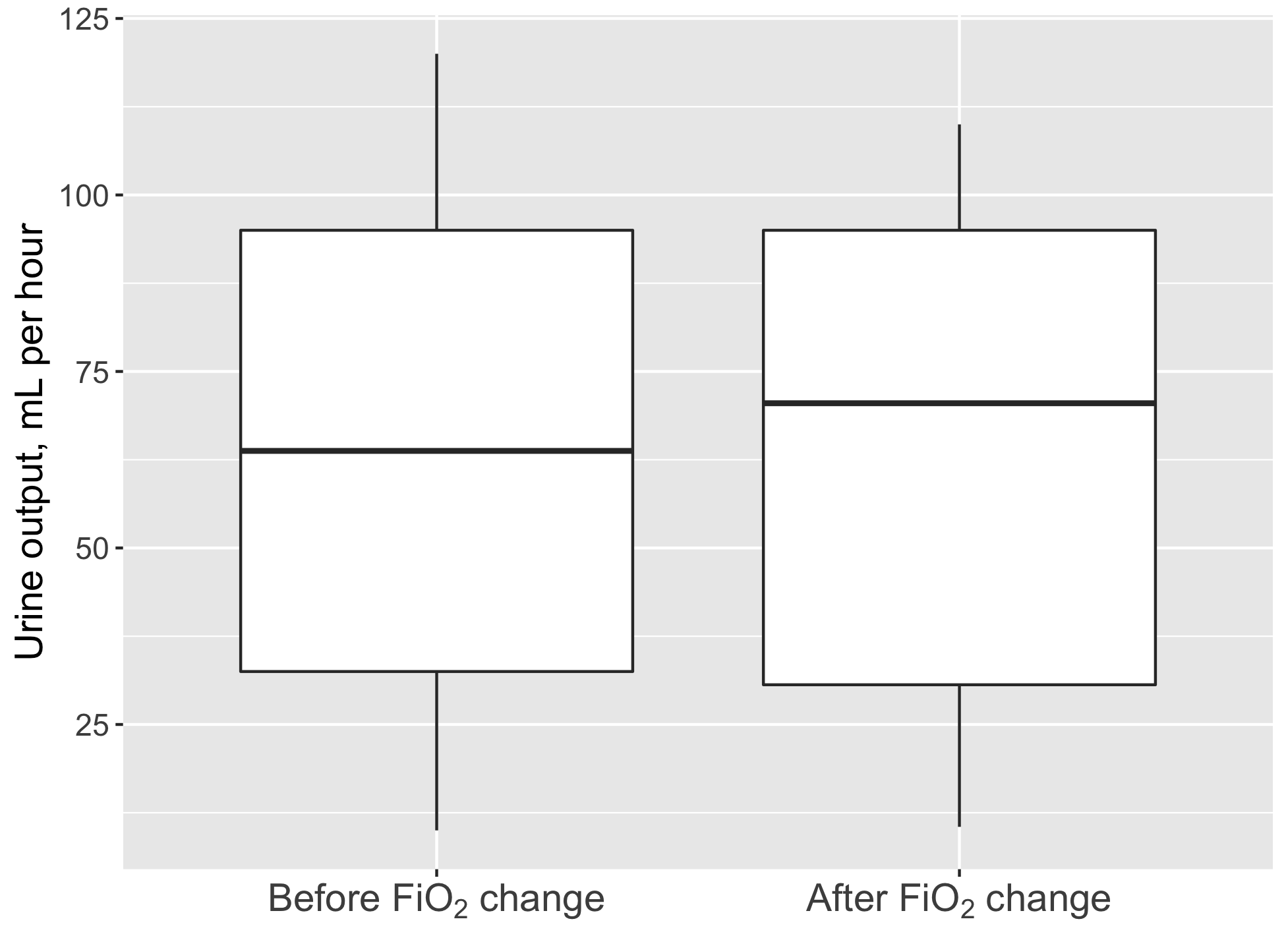

Supplement: Supplementary file 2 — Additional file 2. Box plot illustrating the hourly urinary output before and after a change in FiO2. [file 40635_2022_479_MOESM2_ESM.png]
